# A Case of Pleomorphic Adenoma and Ductal Carcinoma In Situ in the Same Mammary Gland

**DOI:** 10.70352/scrj.cr.24-0100

**Published:** 2025-02-08

**Authors:** Shiho Nagasawa, Koshi Matsui, Misato Araki, Emi Kanaya, Kohji Takagi, Ryo Muranushi, Yoshihiro Shirai, Toru Watanabe, Takeshi Miwa, Katsuhisa Hirano, Shinichi Sekine, Kazuto Shibuya, Isaya Hashimoto, Isaku Yoshioka, Kenichi Hirabayashi, Tsutomu Fujii

**Affiliations:** 1 Department of Surgery and Science, Faculty of Medicine, Academic Assembly, University of Toyama, Toyama, Japan; 2 Department of Diagnostic Pathology, Faculty of Medicine, Academic Assembly, University of Toyama, Toyama, Japan

**Keywords:** pleomorphic adenoma, breast cancer, ductal carcinoma in situ, cartilaginous myxomatous stroma

## Abstract

**INTRODUCTION:**

Pleomorphic adenoma is a benign tumor that frequently occurs in the salivary glands; however, it occurs in the breast rarely. There have been few reports of breast cancer complicated by pleomorphic adenoma of the mammary gland.

**CASE PRESENTATION:**

A 70-year-old woman was found to have a mass lesion in her left breast during a medical examination. A needle biopsy was performed, and a diagnosis of pleomorphic adenoma was made. We performed a partial mastectomy with a margin of several millimeters from the tumor. Pathological examination revealed a diagnosis of pleomorphic adenoma with ductal carcinoma in situ. The resection margin was sufficient, and the patient was followed up.

**CONCLUSIONS:**

Pleomorphic adenoma arising in the mammary gland is difficult to differentiate from adenomyoepithelioma, mucocele-like tumor, and metaplastic carcinoma. Since the tumor can become malignant, resection with a narrow margin is recommended, along with special efforts not to damage the capsule at diagnosis.

## Abbreviations

BI-RADS
: breast imaging reporting and data system
DCIS
: ductal carcinoma in situ
ER
: estrogen receptor
HER2
: human epidermal growth factor receptor-2
PgR
: progesterone receptor

## INTRODUCTION

Pleomorphic adenoma is a benign tumor that predominantly affects the salivary glands while rarely occurring in the breast. There have been few reports of breast cancer in the setting of pleomorphic adenoma of the mammary gland. We report a case of pleomorphic adenoma of the mammary gland with ductal carcinoma in situ (DCIS).

## CASE PRESENTATION

**Fig. 1:**
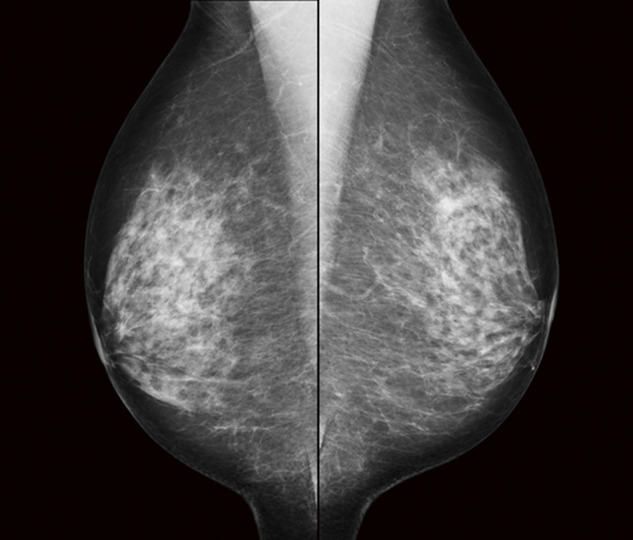
Mammography revealed focal asymmetric density in the left breast.

The patient was a 70-year-old woman with no chief complaint. Her medical history included dyslipidemia, and her family history was unremarkable. She was diagnosed with pleomorphic adenoma of the mammary gland by needle biopsy performed by a local doctor. She was referred to our department for further examination and treatment. Mammography revealed an asymmetric shadow in the left U (**Fig. 1**). Breast imaging reporting and data system (BI-RADS) classification is category 3.

**Fig. 2:**
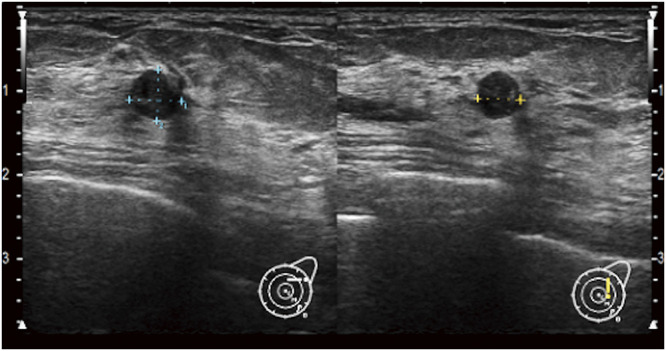
Ultrasound revealed a 6 mm, smooth, well-defined mass in the left C area.

**Fig. 3:**
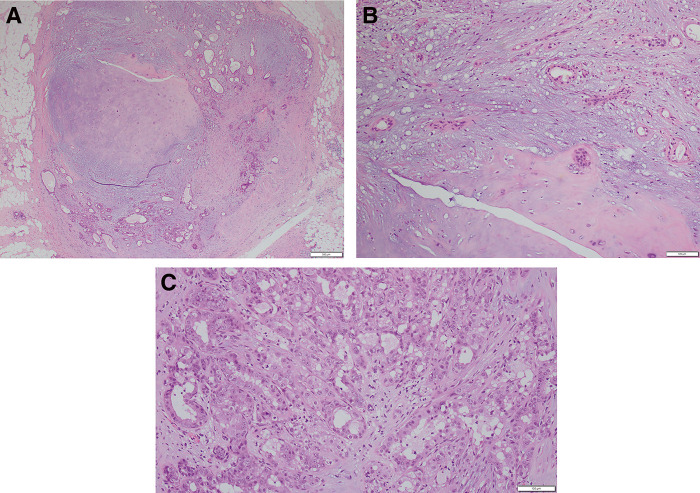
(**A**) Histological imaging. Hematoxylin–eosin stain (×20 at original magnification). This specimen shows a mixture of pleomorphic adenoma and DCIS. (**B**) Histological imaging. Hematoxylin–eosin stain (×100 at original magnification). In the background of myxochondromatous mesenchymal-like elements, loosely arranged spindle cells and ducts lined by cuboidal cells were observed. (**C**) Histological imaging. Hematoxylin–eosin stain (×100 at original magnification). Intraductal epithelial proliferation with a solid-to-cribriform pattern. Nuclear atypia was evident, and atypical mitosis was observed. DCIS, ductal carcinoma in situ

Ultrasonography revealed a 6 mm-long, smooth, well-defined mass in the left C area. There was no evidence of hypervascularization or increased blood flow (**Fig. 2**). BI-RADS classification is category 3. A needle biopsy showed a broad cartilaginous matrix and epithelial components with slightly acidophilic cytoplasm, accompanied by glandular duct formation. Based on these findings, a diagnosis of pleomorphic adenoma was made. The patient was treated with tumor resection to differentiate between metaplastic carcinoma and mucinous carcinoma. Partial mastectomy was performed under local anesthesia with a margin of several millimeters from the tumor. Postoperative pathological examination revealed tubular to cord-like epithelial components against a background of chondromyxoid stroma with cartilage formation and no intraductal papilloma component. The diagnosis of pleomorphic adenoma with DCIS was made based on the presence of intraductal proliferative lesions, some of which were composed of highly atypical cells with atypical mitosis (**Fig. 3**). The size of pleomorphic adenoma is 7 × 6 × 5 mm, and the closest margin is 2 mm. The size of DCIS is 6 × 3 × 2 mm, intermediate-grade DCIS, and the closest margin is 2 mm.

**Fig. 4:**
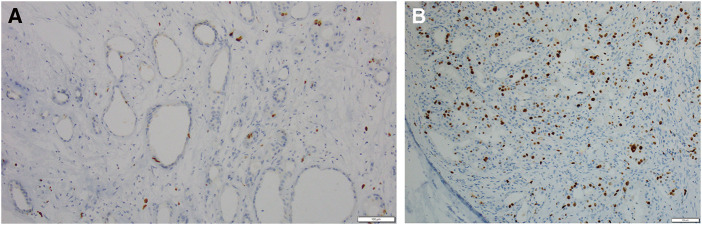
(**A**) Immunohistochemistry Ki-67 antibody in pleomorphic adenoma (×100 at original magnification). (**B**) Immunohistochemistry Ki-67 antibody in DCIS (×100 at original magnification). DCIS, ductal carcinoma in situ

Immunostaining revealed estrogen receptor (ER) (−), progesterone receptor (PgR) (−), human epidermal growth factor receptor-2 (HER2) (0–1+), and Ki-67 (<3%) expression in the pleomorphic adenoma region, and ER (−), PgR (−), HER2 (2+), Ki-67 (15%), and AR (+) in the DCIS region (**Fig. 4**). Since the activities of Ki-67 are quite different, we considered the two to be distinct lesions. We decided that additional resection and irradiation were unnecessary because the DCIS was small, and the margins were negative. We followed up with the patient. One year after surgery, no recurrence was observed.

## DISCUSSION

Pleomorphic adenomas are benign tumors that predominantly occur in the salivary glands and account for 55%–70% of all salivary gland tumors.^1)^ Although the mammary glands are histologically similar to the salivary glands, primary pleomorphic adenomas of the mammary gland are extremely rare. Pleomorphic adenomas are thought to arise from intraductal papilloma.^2)^ On mammography, they are often depicted as masses with clear borders and uniform density, but they may present with findings suspicious of malignancy, such as microcalcifications or masses with indistinct borders. Ultrasonography has revealed a well-defined, smooth, or lobulated, internally homogeneous mass with often enhanced posterior echogenicity.^3)^ Pathologically, there is a mixture of epithelial and mesenchymal components within the tumor and a variety of histologic features, including adenoductal, myoepithelial, myxoid-like, and cartilaginous components.^1)^ Therefore, it is not uncommon for a needle biopsy to be diagnosed as a malignant lesion and a postoperative surgical specimen to be diagnosed as a benign tumor, resulting in overdiagnosis. Primary pleomorphic adenomas of the mammary gland tend to be located around the nipple,^4)^ and cases of recurrence^5)^ and lymph node metastasis^6)^ have been reported.

Ahmad et al. reviewed 77 patients with pleomorphic adenoma; some patients had mastectomies, and most underwent lumpectomy.^7)^ It is important to avoid rupture of the capsule to prevent recurrence.^8)^ If the capsule is damaged, local recurrence may occur due to the seeding of tumor cells. Follow-up is recommended for at least 5 years.^5)^

Salivary gland tumors and mammary gland tumors are morphologically similar, and salivary gland-like tumors of the breast have been reported.^9–11)^ There have been reports of cases in which pleomorphic adenomas and invasive ductal carcinoma of the breast were found in different breasts,^12)^ reports investigating biomarkers in 8 patients with simultaneous or heterochronic salivary gland pleomorphic adenoma and invasive breast cancer,^13)^ and cohort studies showing that salivary gland cancer and pleomorphic adenomas slightly increased the risk of developing breast cancer.^14)^

Reports of breast cancer in the setting of pleomorphic adenoma of the mammary gland are extremely rare. Therefore, there are no classification criteria. On the other hand, carcinomas derived from pleomorphic adenomas of the salivary glands are classified as intraepithelial carcinoma, minimally invasive carcinoma, or extensively invasive carcinoma. Malignant transformation of salivary gland pleomorphic adenomas is more likely to occur in pleomorphic adenomas that have been neglected for a long time or have recurred repeatedly.^15)^ The malignant transformation of pleomorphic adenomas of the salivary glands has been suggested to be associated with molecular alterations such as the overexpression of HER2 protein and androgen receptor expression.^16,17)^ Scarini et al. reported that genetically, *PLAG1* and *HMG2* gene expression is the most common genetic event in pleomorphic adenomas and carcinomas of salivary gland origin.^18)^ Ma et al. reported the involvement of the *TRPS1-PLAG1* fusion gene in the malignant transformation of mammary pleomorphic adenomas.^19)^ This represents a small number of related reports, and further genetic studies are needed. Hayes et al. reported 3 cases of carcinoma from pleomorphic adenoma of the mammary gland, two of which had grade-3 invasive ductal carcinoma and areas of high-grade metaplastic carcinoma with chondroid matrix. One of which had pure high-grade matrix-producing metaplastic carcinoma. Also, a few ducts adjacent to the tumor contained high-grade solid in situ carcinoma. In their article, the distinction between carcinoma ex-pleomorphic adenoma and carcinoma of matrix-producing type was discussed. It was difficult to confirm that they were carcinoma ex-pleomorphic adenoma because there was no long history of a pre-existing stable nodule showing sudden onset of growth, and two of the three had no HER-2 neu amplification. However, the exquisite organization of myoepithelial cells at the periphery of ductal structures in the benign pleomorphic adenoma component was observed; Hayes et al. believe that they were carcinoma ex-pleomorphic adenoma.^20)^

In this case, we observed intermediate-grade DCIS in close pleomorphic adenoma. There was no intraductal papilloma component. It was difficult to distinguish between carcinoma derived from a pleomorphic adenoma and carcinoma adjacent to it, but the possibility that DCIS may have developed from pleomorphic adenoma is suggested by the fact that pleomorphic adenoma may increase the risk of developing breast cancer and that pleomorphic adenoma-derived carcinoma has been reported. As in the aforementioned article, there was no evidence of invasive carcinoma; it may be a preliminary stage from pleomorphic adenoma to invasive carcinoma. The resection margin was adequate; the patient did not receive chemotherapy or radiation therapy and was recurrence-free for 1 year.

## CONCLUSIONS

We encountered a rare case of pleomorphic adenoma of the mammary gland with ductal carcinoma in situ.

## ACKNOWLEDGMENTS

We thank Ichiro Maeda (Department of Pathology, Kitasato University School of Medicine). We also thank all the people who contributed to this work.

## DECLARATIONS

### Funding

The authors received no financial support for the preparation of this case report.

### Authors’ contributions

SN and KM conceived and designed the case report and wrote the main body of the manuscript. KT and KH evaluated the biopsy and resected specimens. EK, RM, YS, TW, TM, KH, SS, KS, IH, IY, and TF participated in the patients’ care and critically reviewed the manuscript. All authors have read and approved the final manuscript. All authors agree to be responsible for all aspects of the study.

### Availability of data and materials

The datasets of this case report are available from the corresponding author upon reasonable request.

### Ethics approval and consent to participate

Not applicable.

### Consent for publication

Informed consent was obtained from the patient for the publication of this case report and accompanying images.

### Competing interests

The authors have no related conflicts of interest to declare.
